# Linking Mesoscale Spatial Variation in Methylmercury Production to Bioaccumulation in Tidal Marsh Food Webs

**DOI:** 10.1021/acs.est.3c04907

**Published:** 2023-11-13

**Authors:** Laurie A. Hall, Isa Woo, Mark Marvin-DiPasquale, John Y. Takekawa, David P. Krabbenhoft, Donald Yee, Letitia Grenier, Susan E. W. De La Cruz

**Affiliations:** † U.S. Geological Survey, Western Ecological Research Center, San Francisco Bay Estuary Field Station, NASA Research Park Bldg. 19, N. Akron Road, Moffett Field, California 94035, United States; ‡ U.S. Geological Survey, Water Mission Area, Earth System Processes Division, 345 Middlefield Road, Menlo Park, California 94025, United States; § U.S. Geological Survey, Mercury Research Laboratory, 8505 Research Way, Middleton, Wisconsin 53562, United States; ∥ San Francisco Estuary Institute, 4911 Central Avenue, Richmond, California 94804, United States

**Keywords:** mercury, methylmercury, sediment biogeochemistry, bioaccumulation, food web, tidal marsh

## Abstract

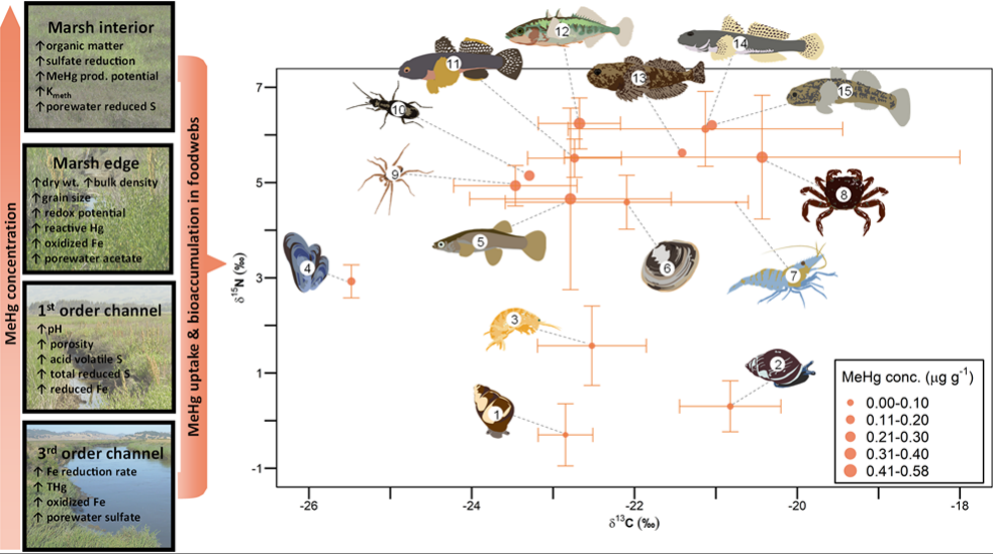

Differences in sediment biogeochemistry among tidal marsh features with different hydrological and geomorphological characteristics, including marsh interiors, marsh edges, first-order channels, and third-order channels, can result in spatial variation in MeHg production and availability. To better understand the link between MeHg production in sediments and bioaccumulation in primary and secondary consumer invertebrates and fish, we characterized mesoscale spatial variation in sediment biogeochemistry and MeHg concentrations of sediments, water, and consumer tissues among marsh features. Our results indicated that marsh interiors had biogeochemical conditions, including greater concentrations of organic matter and sulfate reduction rates, that resulted in greater MeHg concentrations in sediments and surface water particulates from marsh interiors compared to other features. Tissue MeHg concentrations of consumers also differed among features, with greater concentrations from marsh edges and interiors compared to channels. This spatial mismatch of MeHg concentrations in sediments and water compared to those in consumers may have resulted from differences in behavior and physiology among consumers that influenced the spatial scale over which MeHg was integrated into tissues. Our results highlight the importance of sampling across a suite of marsh features and considering the behavioral and physiological traits of sentinel taxa for contaminant monitoring studies.

## Abstract 2

This study reports a spatial mismatch between MeHg bioaccumulation in consumers and MeHg concentrations in sediments and water of tidal marshes, with implications for contaminant monitoring, remediation, and wetland restoration.

## Introduction

Tidal marshes are some of the most productive and economically valuable ecosystems in the world.^1,2^ Their complex food webs span the terrestrial–aquatic interface and support numerous species that are important for commerce, recreation, and subsistence including bivalves, fish, and waterfowl.^3−5^ Concomitantly, tidal marshes are globally significant sites of methylmercury (MeHg) production. Within marshes, this toxic organometallic contaminant is primarily produced in sediments and biomagnifies in food webs, threatening the health of humans and wildlife.

Physical, biogeochemical, and ecological processes occur at different spatial scales within tidal marshes, influencing the production, uptake, and biomagnification of MeHg. Given this complexity, bioaccumulation of MeHg in consumer tissues can vary substantially within and among marshes and is thought to be strongly influenced by the availability of MeHg at the base of the food web (e.g., Rumbold et al.,^6^ Fonseca et al.,^7^ Hall et al.,^8^ Riva-Murray et al.^9^). Biogeochemical conditions in tidal marshes are generally conducive to the MeHg production. For example, organic matter content and sulfate availability can influence the availability of inorganic mercury to methylating microbes and stimulate microbial methylation.^10−15^ However, variation in marsh hydrology and geomorphology can lead to differences in MeHg production and availability among marsh features, including the interior marsh plain (marsh interior), the edge of the marsh plain along channel berms (marsh edge), and channels. Channel geomorphology can be characterized by a hierarchical stream order, ranging from narrow first-order channels in the marsh interior that drain completely at low tide to wider third- or higher-order channels that retain water during low tide and connect the marsh to adjacent waterways.^16^ Sediments in marsh interiors and first-order channels are expected to have biogeochemical conditions, including formation of the oxic–anoxic interface near the sediment surface, that support greater MeHg production and availability compared to marsh edge and third-order channel sediments.^13,17−19^ While previous studies have characterized differences in sediment biogeochemistry and MeHg concentrations among marsh features, few studies have assessed the importance of these differences in explaining variation in bioaccumulation of MeHg in tidal marsh consumers.^13,17−19^

Primary and secondary consumers, such as arthropods, arachnids, mollusks, and fish, are important links in the trophic transfer of MeHg, and their MeHg concentrations are often measured to assess the potential risk of toxicity to higher trophic level consumers, including humans.^8,9,20^ A better understanding of the link between MeHg production in sediments and MeHg bioaccumulation in tidal marsh primary and secondary consumers can improve the design of contaminant monitoring, remediation, and wetland restoration efforts, which often fail to adequately sample the complex suite of hydrological and geomorphological features in tidal marshes. To advance this goal, we characterized mesoscale variation in sediment biogeochemistry and MeHg concentrations of sediments, water, and consumer tissues among marsh features. We controlled for variation in consumer tissue MeHg concentrations related to the trophic position and dietary carbon source using stable carbon and nitrogen isotope values. We hypothesized that biogeochemical conditions of marsh interiors and first-order channels would be more conducive to MeHg production (e.g., greater rates of sulfate and iron reduction and lower concentrations of oxidized species), resulting in greater MeHg concentrations in sediments, water, and consumer tissues compared to marsh edges and third-order channels.

## Materials and Methods

### Study Area

A legacy of mercury contamination from historic mining operations in Northern California has impacted the San Francisco Bay Estuary (SF Bay).^21−23^ Tidal marshes along the Petaluma River in northern SF Bay have some of the highest MeHg concentrations in the region.^24,25^ Our study was conducted at three tidal marshes: Gambinini Marsh, Mid-Petaluma Marsh, and Black John Slough, situated from North to South along a salinity gradient on an approximately 10 km stretch of the Petaluma River (Figure S1). Gambinini Marsh was located <1 km downstream of a water recycling facility that discharged treated freshwater into the Petaluma River and had the lowest mean annual salinity of 12.8 practical salinity units (PSU).^26^ Mid-Petaluma Marsh was located approximately 3 km south of Gambinini Marsh and had a mean annual salinity of 14.4 PSU.^26^ Black John Slough was the farthest south, located near the mouth of the Petaluma River, with the greatest mean annual salinity of 15.3 PSU.^26^ Mean elevations were greater than mean high water at all three marshes, though the elevation of Black John Slough was slightly lower than Gambinini and Mid-Petaluma Marshes.^26^ Thus, marsh plains were inundated regularly when water levels exceeded the mean high water. A previous study determined that sediment concentrations of total mercury (THg) and tin-reducible reactive mercury (RHg), a methodologically defined fraction used to approximate the pool of inorganic Hg(II) available for methylation,^27^ did not differ significantly among these three marshes; however, differences in sediment biogeochemistry, in particular, sediment organic content and porewater Fe(II) concentrations, were linked to differences in MeHg concentrations in sediments and consumers among these marshes.^8^ While controlling for differences among marshes, the current study expands on these findings by examining spatial variation in sediment biogeochemistry and associated MeHg concentrations in sediments, water, and consumer tissues among marsh features with different hydrological and geomorphological characteristics. Additional descriptions of the study area can be found in Tsao et al.^28^ and Hall et al.^8^

### Sample Collection

We measured sediment biogeochemistry and MeHg concentrations in sediments, water, and consumers from four features in each marsh: marsh interiors, marsh edges, first-order channels, and third-order channels. Composited surface (0–2 cm) sediment samples were collected by hand with a 4 cm diameter polycarbonate coring ring in each feature during an ebbing tide in spring (April) and summer (August) of 2006 (*n* = 44).^29^ Samples were collected during spring and summer because previous studies detected seasonal variation in sediment and surface water mercury speciation driven by seasonal hydrologic patterns in SF Bay (i.e., wet winter and spring and dry summer and fall) that could influence MeHg production and availability to food webs.^24,30,31^ Composited samples were collected from exposed sediments in marsh interiors, marsh edges, and first-order channels, whereas samples from third-order channels were collected at a very low tide when minimal water was present. Composited samples were generated from marsh interiors by combining subsamples collected every 0.67 m along 1.5 m diameter circular plots and from marsh edges, first-order channels, and third-order channels by combining subsamples collected every 1 m along 7 m transects that ran parallel to the channel edge. Transects for marsh edges were located along the berms of first- and third-order channels, where the channel bank meets the marsh plain. Channel transects were located along the center of the channels, approximately midway between banks. Samples were collected in an upstream direction to minimize the disturbance of consecutive subsamples. Composited samples were stored chilled in mason jars with minimal to no headspace for approximately 24 h before porewater extraction and subsampling for laboratory analyses in an N_2_ flushed anaerobic environment. Porewater was collected from each composited sediment sample under oxygen-free conditions by centrifugation and filtered to 0.45 μm. Surface water (<10 cm) was collected from pools of standing water that remained present in marsh interiors throughout the tidal cycle and from first- and third-order channels during a high slack tide in April and August of 2006 (*n* = 36). Half of each water sample was filtered to 0.45 μm to measure the filterable fraction of MeHg. Additional details about sediment and water sampling are described in Yee et al.^19^

Primary and secondary consumers from 15 genera were collected during their peak abundances (May–August) from the four marsh features along the same transects used for sediment and water collection.^29^ Gastropods and bivalves (genera: *Assiminea, n* = 6; *Myostotella*, *n* = 6; *Geukensia*, *n* = 19; and *Macoma*, *n* = 11) were collected by hand. Amphipods (genus: *Traskorchestia*, *n* = 12) were collected using 15 mL plastic bottles with moistened coffee filters placed along transects for 2 days.^8,19^ Beetles and spiders (genera: *Bembidion*, *n* = 2; and *Pardosa*, *n* = 11) were collected using a small handheld vacuum with a fine mesh sieve.^8,19^ Shrimps and crabs (genera: *Palaemon*, *n* = 3; and *Hemigrapsus*, *n* = 6) were collected with a hand-net and in minnow traps. Fish (genera: *Tridentiger*, *n* = 12; *Acanthogobius*, *n* = 5; *Gillichthys*, *n* = 1; *Gambusia*, *n* = 5; *Gasterosteus*, *n* = 16; *Cottus*, *n* = 1) were collected in minnow traps. We limited fish collections so that the range in fish lengths was ≤30 mm for each species because concentrations of THg can increase as a function of fish length and weight; however, previous studies in SF Bay and elsewhere detected weak or no relationship between THg concentrations and fish length in some of the taxa included in our study.^32−34^ Following collection, consumers were kept alive in a refrigerator at 4 °C for 24 h to purge their gut contents and then frozen at −20 °C. Fish were euthanized by percussive stunning, followed by freezing. Prior to analysis, consumers were thawed and rinsed three times with distilled water. Gastropods, bivalves, shrimp, and crabs were deshelled. Consumers were dried to a constant mass, and multiple individuals from each genus were combined into composited samples (marsh interior *n* = 20, marsh edge *n* = 17, first-order channel *n* = 29, third-order channel *n* = 50) to achieve a dry mass of 1.0 mg for stable isotope analysis, 100 mg for MeHg analysis of invertebrate consumers, and 50 mg for THg analysis of fish. We assumed THg measured in fish was equivalent to MeHg because previous studies have shown that a majority (80–100%) of THg in fish muscle was MeHg.^35,36^

### Laboratory Analyses

We measured and calculated 26 variables related to sediment and porewater biogeochemistry including sediment concentrations of THg, inorganic RHg, MeHg, acid volatile sulfur (AVS), total reduced sulfur (TRS), acid extractable ferrous iron (Fe(II)), amorphous ferric iron (Fe(III)_a_), and crystalline ferric iron (Fe(III)_c_); percent organic matter of the sediment assessed by loss on ignition (LOI); MeHg production rate constant (*k*_meth_); MeHg production potential (MPP); sediment redox potential (Eh), pH, grain size (GS), percent of wet weight (DW), bulk density (BD), and porosity (POR); sediment microbial rates of sulfate reduction (SR), iron reduction (Fe(II) rate), and methane production (CH_4_ prod.); and porewater concentrations of dissolved organic carbon (pwDOC), acetate (pwAcetate), Fe(II) (pwFe(II)), sulfide (pwH_2_S), sulfate (pwSO_4_^2–^), and chloride (pwCl^–^). In surface water, we measured concentrations of filterable and particulate MeHg. All measurements were conducted following established protocols described in the Supporting Information (Section 1).

Sediments were analyzed for THg and MeHg concentrations, and water (both filtered and unfiltered) was analyzed for MeHg concentrations at the U.S. Geological Survey (USGS) Mercury Research Laboratory (Middleton, WI). All other measurements were conducted at the USGS Biogeochemistry Laboratory (Menlo Park, CA). Analytical duplicates, matrix spike recoveries, and certified reference materials appropriate for each assay were included with all analytical batches for quality control as described in the Supporting Information (Section 1). Measurements from digestion blanks that used all reagents in an analysis were subtracted from the final sample measurements. Field blanks, collected during each sampling event, were analyzed to provide quality assurance of the sampling and laboratory protocols. The Standard Reference Material IAEA 405 was used for mercury analysis in sediments, and recoveries were within ±10% of the certified value. No certified reference material exists for mercury in water at environmentally relevant concentrations; therefore, a commercially available mercury standard was verified against a certified National Institute of Standards and Technology (NIST) standard for mercury analysis in water. Replicates were within ±20% agreement. Detailed protocols for laboratory analyses and quality control can be found in the Supporting Information (Section 1).

Analyses of δ^13^C and δ^15^N values of whole consumers were conducted at the Northern Arizona University Colorado Plateau Stable Isotope Lab (Flagstaff, AZ). Samples were packed into tin capsules, and isotope ratios were measured with a continuous-flow ThermoFinnigan Deltaplus Advantage gas isotope ratio mass spectrometer (Waltham, MA) interfaced with a Costech ECS 4010 elemental analyzer (Valencia, CA). The long-term analytical precision was ±0.1‰ for δ^13^C and ±0.2‰ for δ^15^N. Vienna Pee Dee Belemnite (VPDB) and air (AIR) were used as standards for δ^13^C and δ^15^N, respectively, and Peach Leaf (NIST 1547) standards and blanks were included with each run to correct for drift. Mean relative percent difference (±standard deviation) for duplicates was 0.5 ± 0.5% for δ^13^C and 0.7 ± 0.8% for δ^15^N. Isotope ratios were reported in parts per thousand (‰) using delta notation with δ^*h*^*N* = (*R*_sample_/*R*_standard_ −1) × 1000, where *R* was the ratio of enriched to depleted isotopes for the sample or standard, *N* was the element of interest, and *h* was the mass of the enriched isotope. Because most samples had C/N ratios >4, we normalized δ^13^C values to account for variable lipid content according to the following equation from Post et al.^37^
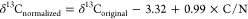


We also applied a baseline correction to δ^15^N values to make values comparable among sites by subtracting the mean δ^15^N value of primary consumers at the base of the food web (i.e., *Assiminea sp*. and *Myosotella myosotis* which had the lowest δ^15^N values) in each marsh from the δ^15^N value of each consumer from the marsh.^38^ Normalized δ^13^C and baseline-corrected δ^15^N values were used in all subsequent analyses.

Invertebrate consumers were analyzed for MeHg at Battelle Marine Sciences Laboratory (Sequim, WA) following the method of Bloom.^39^ MeHg standards were made from pure stock methylmercuric chloride (Alfa Aesar, Ward Hill, MA) and calibrated against certified reference materials (NRCC DORM-2, DOLT-2; National Research Council of Canada, Ottawa, Canada). Quality assurance included the analysis of certified reference materials, system blanks, and sample duplicates. Mean relative percent difference for duplicates (±standard deviation) was 7.5 ± 5.0%, and mean recoveries of certified reference material and matrix spikes were 98.3 ± 11.7 and 105.0 ± 9.2%, respectively. Concentrations of MeHg were reported in μg g^–1^ dry mass.

Whole fish were analyzed for THg at the USGS Davis Field Station Mercury Laboratory (Davis, CA) with a Milestone DMA-80 Direct Mercury Analyzer (Milestone Inc., Monroe, CT) following U.S. Environmental Protection Agency Method 7473 described in Ackerman et al.^40,41^ Certified reference materials (NRCC DORM-2, TORT-2; National Research Council of Canada, Ottawa, Canada) were used as calibration standards. Quality assurance included analysis of certified reference materials, system blanks, and sample duplicates. The mean relative percent difference for duplicates (±standard deviation) was 3.6 ± 2.4%, and mean recoveries of certified reference material and matrix spikes were 110.0 ± 9.6 and 113.3 ± 19.9%, respectively. Concentrations of THg were reported in μg g^–1^ dry mass.

### Statistical Analyses

All statistical analyses were run in R v. 4.0.2.^42^ We characterized biogeochemical conditions in sediments and porewater from different marshes and features using a principal component analysis (PCA) with the *rda* function in the Vegan package.^43^ Biogeochemical variables were scaled and centered prior to PCA. Differences in MeHg concentrations of sediments and water among marsh features were assessed with General Linear Models (GLMs) using the *lm* function followed by Tukey’s HSD Multiple Comparisons tests (MCTs) in the multcomp package.^42,44^ Site was included in GLMs to account for variation among marshes in environmental conditions such as salinity, which has been shown to influence stable isotope values of consumers.^45^

We plotted δ^15^N values as a function of δ^13^C values for primary and secondary consumers to visualize variation in trophic positions and dietary carbon sources in the marsh food web. Then, we evaluated differences in tissue MeHg concentrations of primary and secondary consumers among marsh features using a GLM and MCT.^42,44^ We included the site, trophic position (measured as δ^15^N values), and dietary carbon source (measured as δ^13^C values) in the GLM to account for variation related to these variables. We used a likelihood ratio test in the lmtest package to compare the likelihood of the full model (Feature + Site + Trophic position + Dietary carbon source) to that of a reduced model (Site + Trophic position + Dietary carbon source).^46^ The full model was used to generate predicted tissue MeHg concentrations with the *lm.predict* function.^42^ For each feature, 300 predicted tissue MeHg concentrations were estimated for δ^13^C and δ^15^N values that were randomly sampled from the observed range of isotope values. Concentrations of MeHg were natural log-transformed in all models.

## Results and Discussion

### Mesoscale Variation in Sediment Biogeochemistry and MeHg Production

**Figure 1:**
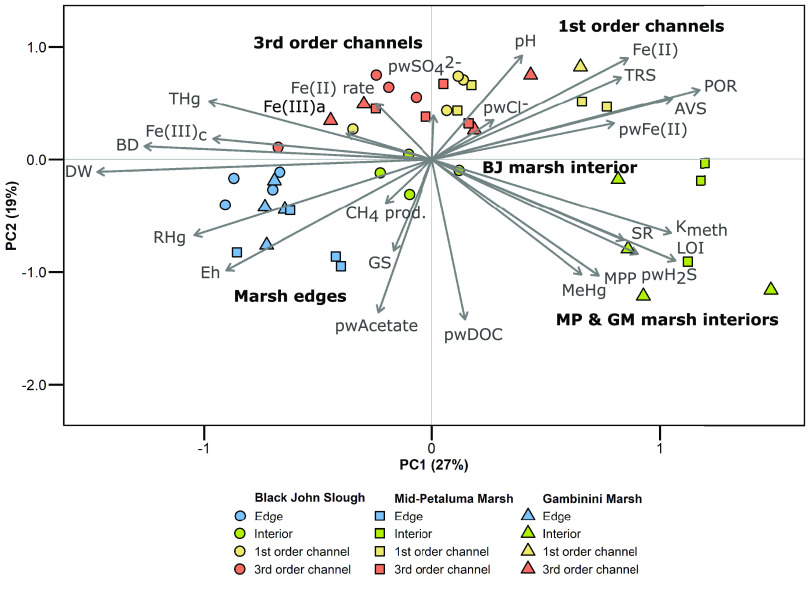
Principal component analysis showing the relationships of 26 biogeochemical variables measured in sediments and porewater from four features sampled at three tidal marshes: Black John Slough (circles), Mid-Petaluma Marsh (squares), and Gambinini Marsh (triangles) along the Petaluma River in California, USA. The percent of variation explained by each principal component is indicated in parentheses. Samples were distributed among five groups: marsh edges; marsh interiors of Mid-Petaluma and Gambinini Marshes; marsh interiors of Black John Slough; first-order channels; and third-order channels. See Laboratory Analyses for variable abbreviations.

In tidal marshes, anoxic sediment conditions combined with high organic matter content and the availability of sulfate and iron stimulate microbial reduction by sulfur-reducing, iron-reducing, and methanogenic bacteria that can result in MeHg production.^10−13,47,48^ Our results indicated sediment biogeochemistry differed among marshes and features (Figure 1 and Table S1). The first principal component of the PCA primarily separated marsh interiors and first-order channels from marsh edges and third-order channels, explaining 27% of variation in sediment biogeochemistry (Figure 1). The second principal component explained 19% of variation in sediment biogeochemistry and separated sediments collected on marsh plains (i.e., interiors and edges) from channels (Figure 1). Sediments were generally distributed among five groups: marsh edges; marsh interiors of Black John Slough; marsh interiors of Mid-Petaluma and Gambinini Marshes; first-order channels; and third-order channels (Figure 1).

**Figure 2:**
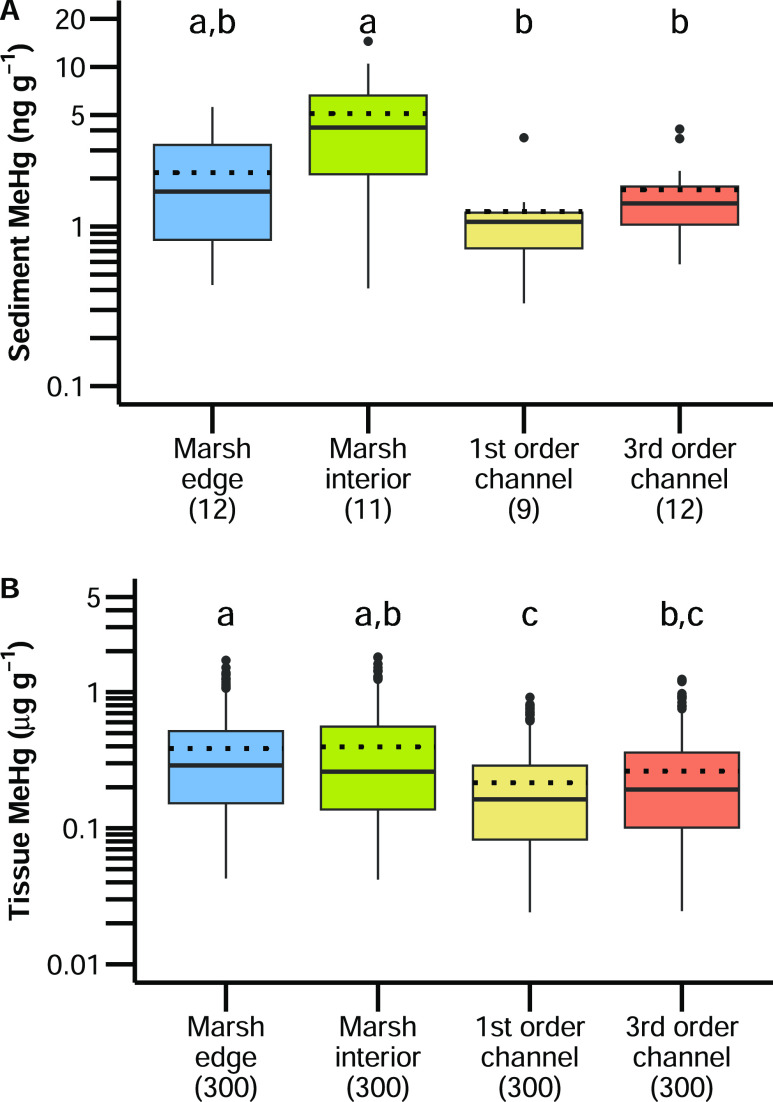
(A) Concentrations of methylmercury (MeHg) in sediments (ng g^–1^) and (B) predicted MeHg concentrations of primary and secondary consumer tissues (μg g^–1^) from four features: edges, interiors, first-order channels, and third-order channels, collected at tidal marshes along the Petaluma River in California, USA. Boxes indicate the first and third quartiles separated by the median (solid line) and mean (dotted line). Whiskers extend to the maximum/minimum observed values or up to 1.5 times the interquartile range. Different letters indicate significant differences based on post hoc Tukey’s HSD tests. Sample sizes are indicated in parentheses.

Sediments from marsh edges were characterized by greater dry weight (DW); greater bulk density (BD); larger grain size (GS); greater *E*_h_ values; and greater concentrations of RHg, porewater acetate (pwAcetate), and crystalline ferric iron (Fe(III)_c_), indicating that sediments from marsh edges were more compact, less saturable, and more oxidized than other features (Figures 1 and 4, and Table S1). In agreement with our hypothesis, these conditions resulted in lower sediment concentrations of MeHg in marsh edges compared to marsh interiors, although these differences were not statistically significant (*p* = 0.09; Figure 2A), perhaps due to our modest sample sizes. Similarly, a previous study that assessed variation in sediment organic content and geochemical indicators of redox conditions along an elevation gradient in the tropics suggested that less organic and more oxidized conditions were generally associated with lower sediment MeHg concentrations.^49^ Our results also indicated that sediment conditions from Black John Slough interiors were more similar to marsh edges and, therefore, less conducive to MeHg production compared to interior sediments from Gambinini and Mid-Petaluma Marshes (Figure 1). This corroborated the findings of a previous study that detected lower sediment MeHg concentrations at Black John Slough compared to Gambinini and Mid-Petaluma Marshes.^8^ In contrast, sediments from marsh interiors of Mid-Petaluma and Gambinini Marshes were characterized by greater percent organic matter (LOI); greater rates of microbial sulfate reduction (SR); greater MeHg production potential (MPP); greater methylation rate constants (*K*_meth_); and greater concentrations of porewater H_2_S (pwH_2_S) and MeHg (Figures 1 and 4). These conditions were indicative of high organic matter and chemical reduction rates and were associated with greater sediment MeHg concentrations compared to those of other features (Figure 2A). Gilmour et al.^13^ obtained similar results; anoxic and reducing sediment conditions were associated with greater MeHg concentrations in the interior of high-elevation tidal marsh compared to other areas of the marsh.

Several hydrological, geomorphological, and ecological characteristics could contribute to differences in sediment biogeochemistry and MeHg production between marsh edges and interiors. Due to complex relationships between marsh topography, sediment grain size, hydroperiod, drainage dynamics, and vegetation composition, surface sediments of marsh edges along channels tend to be more elevated (due to berm formation), to drain faster, to be more oxidized, and to have lower organic content compared to marsh interiors.^50−55^ In contrast, once inundated, marsh interiors stay wetter for longer, allowing surface sediments to become more chemically reducing during saturation.^52−55^ Differences in redox conditions between marsh edges and interiors may partially explain the higher MeHg concentrations (favored under reducing conditions) in marsh interiors and the lower MeHg concentrations (typical under oxic conditions) in marsh edges. Further, cycles of wetting and drying have been shown to affect MeHg production, with dry periods facilitating the reoxidation of reduced forms of sulfur and iron (e.g., FeS, FeS_2_, and free H_2_S), and rewetting periods stimulating the activity of Hg(II)-methylating sulfate (SO_4_^2–^) and Fe(III) reducing microbes that can utilize those reoxidized species.^56−58^ Thus, the longer saturation periods associated with marsh interiors may also facilitate more reducing conditions and more time favoring MeHg production compared to marsh edges. In addition, root biomass influences the organic content of sediments because plants exude labile organic carbon through their roots where it can be metabolized by methylating microbes.^13,59,60^ In SF Bay tidal marshes, pickleweed (*Salicornia pacifica*), which has a high root biomass, was the dominant plant in marsh interiors and was present at 97–99% of vegetation points surveyed in our marshes.^19,26,59^ In contrast, plants such as gumplant (*Grindelia stricta*) and big saltbush (*Atriplex lentiformis*) were less common than pickleweed, occurring at less than 15% of vegetation points surveyed in our marshes.^19,26^ Despite the lower overall abundance of these species, they commonly occurred along marsh edges and may have had different root biomasses compared to pickleweed.

**Figure 3:**
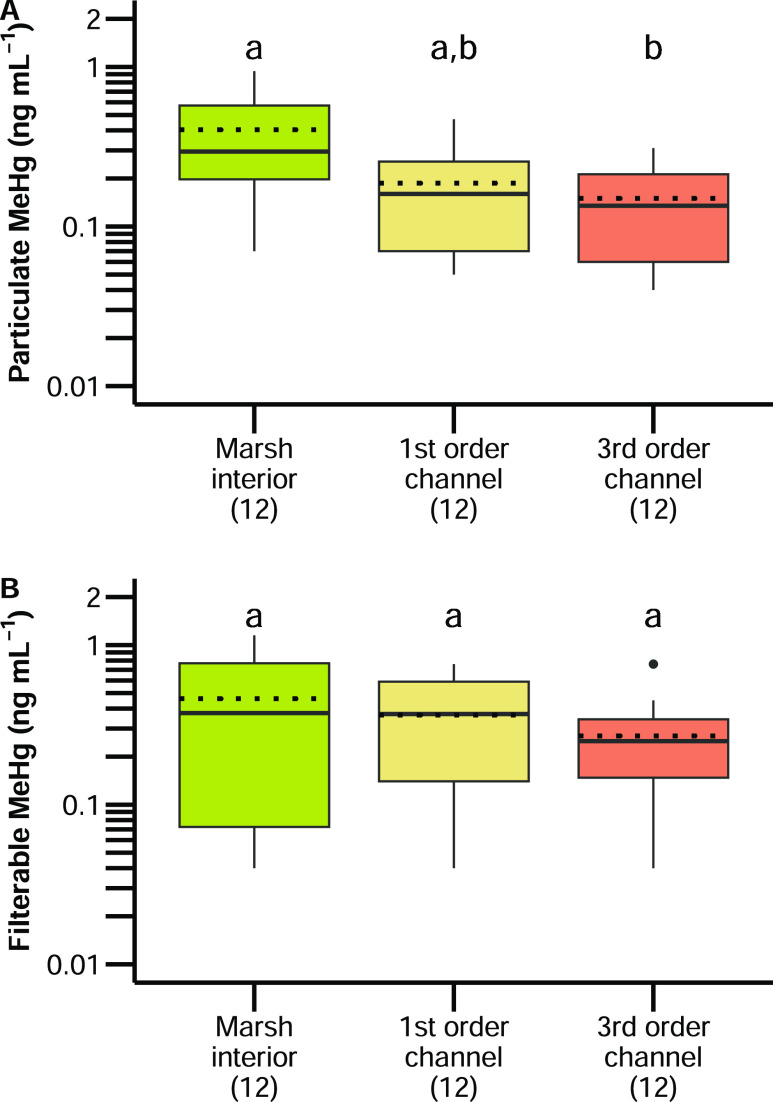
Concentrations of (A) particulate (ng mL^–1^) and (B) filterable (ng mL^–1^) methylmercury (MeHg) in surface waters from three features: interiors, first-order channels, and third-order channels, collected at tidal marshes along the Petaluma River in California, USA. Boxes indicate the first and third quartiles separated by the median (solid line) and mean (dotted line). Whiskers extend to the maximum/minimum observed values or up to 1.5 times the interquartile range. Different letters indicate significant differences based on post hoc Tukey’s HSD tests. Sample sizes are indicated in parentheses.

**Figure 4:**
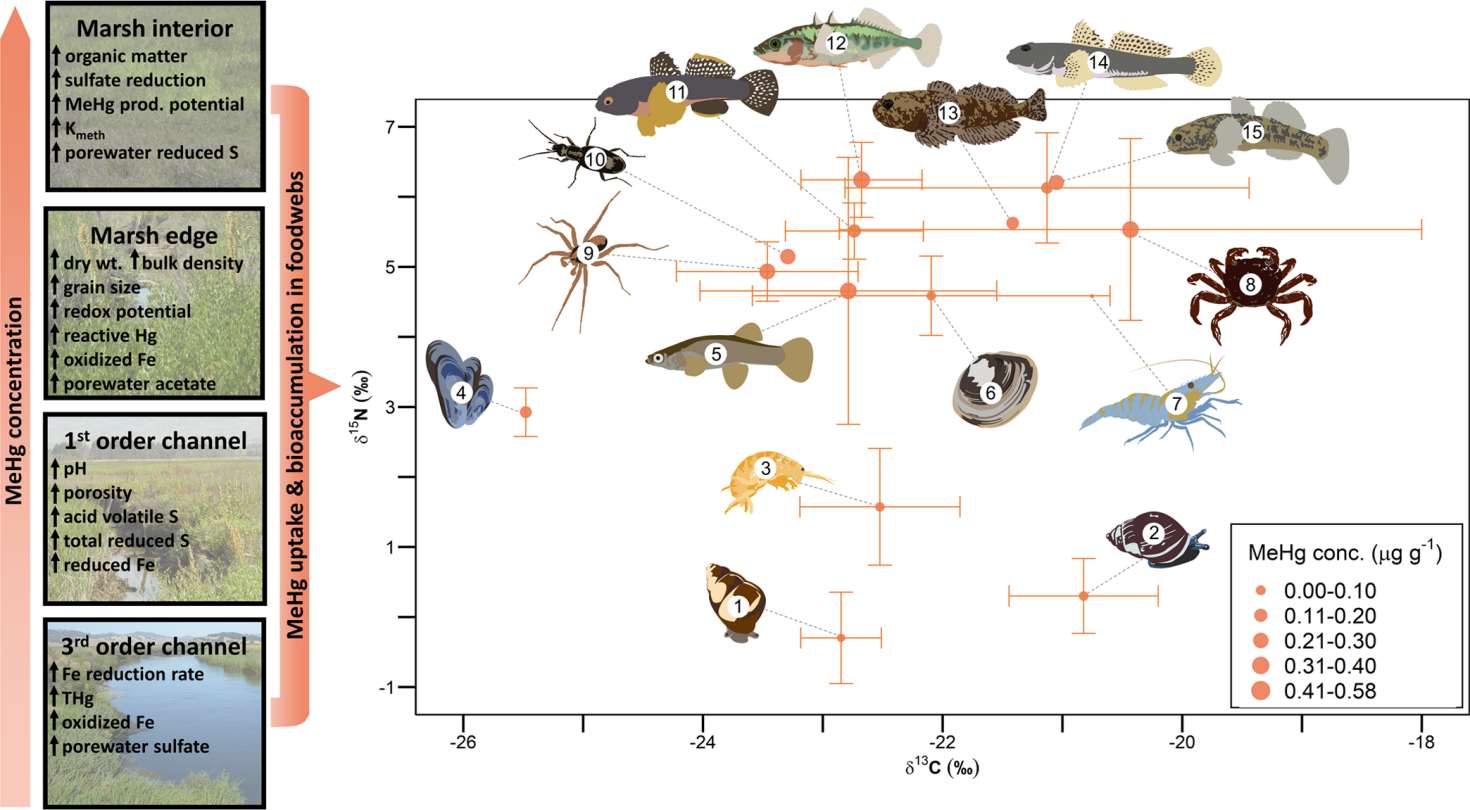
Summary of sediment biogeochemical conditions and MeHg concentrations in four marsh features and mean (±95% confidence intervals) stable δ^13^C (normalized for variable lipid content) isotope values, δ^15^N (baseline-corrected) isotope values, and methylmercury (MeHg) concentrations of primary and secondary consumers in tidal marsh food webs collected at three marshes: Black John Slough, Mid-Petaluma Marsh, and Gambinini Marsh, along the Petaluma River in California, USA. Consumer taxa include: (1) *Assiminea sp.*, (2) *M. myosotis*, (3) *Traskorchestia sp.*, (4) *G. demissa*, (5) *Gambusia affinis*, (6) *Macoma petalum*, (7) *Palaemon sp.*, (8) *Hemigrapsus sp.*, (9) *Pardosa sp.*, (10) *Bembidion sp.*, (11) *Tridentiger bifasciatus*, (12) *Gasterosteus aculeatus*, (13) *Cottus gulosus*, (14) *Acanthogobius flavimanus*, and (15) *Gillichthys mirabilis*.

Our results also indicated that biogeochemical conditions in surface sediments of first-order channels were more chemically reduced compared to third-order channels. Specifically, first-order channel sediments had greater pH; greater porosity (POR); and greater concentrations of total reduced sulfur (TRS), acid volatile sulfur (AVS), acid extractable ferrous iron (Fe(II)), and porewater ferrous iron (pwFe(II); Figures 1 and 4, and Table S1). In contrast, third-order channel sediments were characterized by greater microbial iron reduction rates (Fe(II) rate) and greater concentrations of THg, amorphous ferric iron (Fe(III)_a_), and porewater sulfate (pwSO4^2–^; Figures 1 and 4, and Table S1). Despite these differences in sediment redox conditions, we did not observe significantly greater MeHg concentrations in sediments from first-order channels compared with third-order channels (*p* = 0.63; Figure 2A). This may have resulted from differences between the two features in the competing processes of MeHg production and degradation that ultimately led to similar steady-state MeHg concentrations. Further, the high frequency of tidal flushing in channels (i.e., twice per day) likely served to keep the MeHg formed in the porewater phase of the surface sediment well flushed and consistent along the length of the channel network. Similarly, the lack of significant differences between channel features in particulate (*p* = 0.87; Figure 3A) and filterable (*p* = 0.71; Figure 3B) MeHg concentrations in surface water may reflect the high frequency of flushing, resulting in more homogeneous MeHg concentrations in the surface water of channels. This aligned with previous research that indicated suspended sediments and associated particulate mercury in SF Bay were homogenized and transported by large-scale lateral advection and mixing driven by winds, tides, and seasonal river inputs.^24,61^

Although we did not detect differences between channel features in surface water particulate or filterable MeHg concentrations, we did detect greater particulate MeHg concentrations in pools of standing water from marsh interiors compared to channel features (Figure 3); however, only the difference in particulate MeHg between marsh interiors and third-order channels was statistically significant (*p* < 0.03; Figure 3A). Greater concentrations of MeHg in sediments and surface water particulates from marsh interiors could be the result of both greater MeHg production and lower inundation frequencies in marsh interiors compared with channel features. Interior sediments of high-elevation tidal marshes, like those in our study, would be inundated only twice per lunar month during spring tides, whereas channels would be flushed twice a day by diurnal tides.

### Mesoscale Variation in MeHg Bioaccumulation

MeHg in sediments and water can be concentrated by several orders of magnitude in primary producers at the base of food webs and subsequently biomagnified in consumers, reaching potentially toxic concentrations for humans and wildlife.^62−64^ Previous studies have demonstrated that differences in MeHg accumulation among food webs were primarily driven by concentrations at the base of food webs, rather than by the relative extent of trophic transfer.^6−9^ This makes primary and secondary consumers that forage at the base of food webs important targets for contaminant monitoring programs. We measured average tissue MeHg concentrations ranging from 0.04 to 0.58 μg g^–1^ among primary and secondary consumer taxa (Figure 4 and Table S2). Few MeHg concentrations have been published for similar taxa of tidal marsh invertebrates, but studies from other aquatic habitats are available for comparison. The MeHg concentrations we observed in amphipods and gastropods were greater than those reported from uncontaminated freshwater and agricultural wetlands.^65,66^ Similarly, we observed MeHg concentrations in the bivalve *Geukensia demissa* that were approximately 10 times greater than those observed in an uncontaminated estuary in Florida, USA.^6^ In contrast, spiders in our marshes had much lower MeHg concentrations than those from an agricultural wetland with mining contamination in China’s Wanshan District.^67^ In fish, the concentrations we observed were within the ranges reported for similar taxa in south SF Bay and a contaminated estuary in Portugal, whereas the concentrations we observed were greater than those from an uncontaminated estuary in Florida, USA and uncontaminated streams in Quebec, Canada.^6,33,34,68^ Information on toxicity benchmarks was limited for the primary and secondary consumers included in our study; however, previous studies have indicated that MeHg bioaccumulation in higher trophic level consumers in SF Bay marshes can exceed no adverse effects limits.^40,69,70^

Our full linear model, which included δ^15^N, δ^13^C, site, and marsh features as predictors, explained 42% of variation in primary and secondary consumer tissue MeHg concentrations, whereas the reduced model that did not include feature explained only 37% of variation. Further, the likelihood of the data was greater for the full model compared to the reduced model (χ^2^ = 12.2, *d*_f_ = −3, *p* < 0.01). Therefore, including feature—a predictor associated with mesoscale spatial variation in tidal marsh hydrology and geomorphology—increased the likelihood of the data and helped explain additional variation in tissue MeHg concentrations of consumers. This suggested that sampling efforts for contaminant monitoring programs could be stratified among features to adequately quantify variation in MeHg bioaccumulation and assess the risk of toxicity to humans and wildlife.

Indeed, model-predicted tissue MeHg concentrations of primary and secondary consumers differed among the marsh features (Figure 2B). Contrary to our hypotheses, tissue MeHg concentrations did not differ significantly between consumers from marsh interiors and edges (*p* = 0.99) nor from first-order channels compared to third-order channels (*p* = 0.81; Figure 2B). Instead, tissue MeHg concentrations were greater in consumers from marsh plains (i.e., edges and interiors) compared to channels, though the difference between marsh interiors and third-order channels was not statistically significant (*p* = 0.08; Figure 2B). The spatial pattern of tissue MeHg concentrations differed from the pattern we observed in sediments and surface water particulates, which generally had greater MeHg concentration in marsh interiors compared to other features (Figures 2 and 3A). This spatial mismatch may have resulted from differences in behavior and physiology of consumers that influenced the spatial scale at which MeHg exposure and uptake occurred.^9,20,71^

Trophic position (measured with δ^15^N stable isotopes) and dietary carbon source (measured with δ^13^C stable isotopes) can help elucidate differences in behavior and physiology that contribute to differences in MeHg bioaccumulation among consumers. Concordant with previous studies, we detected a significant increase in tissue MeHg concentrations of primary and secondary consumers with increasing δ^15^N values (*p* < 0.01; Figure S2) and decreasing δ^13^C values (*p* < 0.01; Figure S3).^64,68,72,73^ The δ^15^N values (baseline-corrected) in our marsh food webs ranged from −0.9 to 7.8‰, and the δ^13^C values (normalized for variable lipid content) ranged from −28.3 to −17.7‰ (Figure 4). These values were within the ranges reported for primary producers in SF Bay, assuming an average trophic fractionation of 3.4‰ for δ^15^N and 0.4‰ for δ^13^C.^74,75^ Primary consumers included two genera of gastropods (*Assiminea sp*. and *M. myosotis*), which had the lowest δ^15^N values, followed by amphipods (*Traskorchestia sp*.) and a bivalve (*G. demissa*; Figure 4). A second species of bivalve (*M. petalum*) had δ^15^N values more similar to those of the secondary consumers, which included spiders (*Pardosa sp.*), beetles (*Bembidion sp.*), crabs (*Hemigrapsus sp.*), shrimp (*Palaemon sp.*), and fish (*T. bifasciatus*, *A. flavimanus*, *G. mirabilis*, *G. affinis*, *G. aculeatus*, and *C. gulosus*; Figure 4). Dietary carbon sources were similar for all taxa, with mean δ^13^C values between −23.5 and −20.4‰, except a bivalve (*G. demissa*) which had a lower mean δ^13^C value of −25.5‰ (Figure 4). The large range of δ^13^C values we observed indicated that primary consumers in SF Bay tidal marshes likely foraged on different types of primary producers, including marine phytoplankton, benthic diatoms, algae, and C3 plants.^75^ In contrast, C4 plants, which have more enriched δ^13^C values than C3 plants, did not contribute largely to primary consumer diets in our study.^75^ This is likely because C4 plants, such as *Distichlis spicata* and *Spartina foliosa*, had much lower coverage at our sites.^26^ In addition, bacterial respiration could influence δ^13^C values at the base of food webs, with increased bacterial respiration in sediments and water linked to depleted δ^13^C values and greater MeHg concentrations in consumers.^68^ Future studies of spatial variation in bacterial respiration among marsh features could help elucidate the relationship between bacterial respiration and MeHg bioaccumulation in tidal marsh consumers.

We detected differences in MeHg concentrations among taxa that could have resulted from differences in behavior and physiology. For example, amphipods had greater MeHg concentrations than gastropods (Figure 4). Amphipods primarily consume detritus which may have greater MeHg concentrations than live plant material on which gastropods forage.^76^ We also detected differences in MeHg concentrations between two species of bivalves (Figure 4); *G. demissa* is primarily a filter feeder, whereas *M. petalum* has a more flexible foraging strategy, switching between filter feeding and deposit feeding.^77^ Despite occupying similar channel features, *G. demissa* had lower δ^15^N and δ^13^C values, indicating that it occupied a lower trophic position and foraged primarily on phytoplankton compared to *M. petalum* that occupied a higher trophic position and foraged on both phytoplankton and detrital deposits (Figure 4).

Movement of consumers can also influence their MeHg exposure and uptake. Bivalves, for example, were sessile, whereas other taxa in our study, including amphipods, gastropods, spiders, beetles, shrimp, crabs, and fish, could move and forage among different tidal marsh features, integrating MeHg at different spatial scales. Our results indicated that consumers from marsh plains had greater tissue MeHg concentrations compared to consumers from channels, supporting the idea that marsh plain consumers bioaccumulate greater MeHg concentrations as they move and forage between marsh edges and interiors compared to channel consumers that bioaccumulate lower MeHg concentrations as they move and forage along the channel network.

In addition, exposure pathways may differ among taxa with different physiologies. For example, bioaccumulation in benthic organisms, such as amphipods, gastropods, and bivalves, can occur via both ingestion and absorption, and the relative exposures through each pathway are likely to differ among taxa with different physiologies.^71,78,79^ Differences in foraging behavior, diet, movement, and physiology influence the spatial and temporal scales over which MeHg produced in sediments is accumulated into consumer tissues. Therefore, these characteristics must be carefully considered when selecting sentinel species for contaminant monitoring programs to ensure that the MeHg bioaccumulation measured in consumer tissues accurately reflects the MeHg availability at a given sampling location.

### Implications for Contaminant Monitoring, Remediation, and Restoration

Tidal marshes have a diverse topology that influences the hydrological and geomorphological characteristics of different features including marsh edges, marsh interiors, first-order channels, and third-order channels.^50−55^ Our results demonstrated that these features have distinct biogeochemical conditions that may have influenced the production and availability of MeHg to the food web (Figures 1 and 4). Tidal marshes are generally considered to be MeHg sinks; however, high spring tides and groundwater discharge can transport MeHg to surrounding areas.^61,80,81^ Therefore, channel density and drainage could strongly influence MeHg production and availability at larger scales, and models that account for variation in sediment biogeochemistry among marsh features could improve estimates of MeHg fluxes.

Given the differences in MeHg production and availability among features, potential strategies for remediation and restoration in tidal marshes could include vegetation management, capping or coagulation of contaminated sediments, and altering marsh hydrology by increasing channelization or routing freshwater over marsh plains. Restoration plans that emphasize channelization and drainage could help reduce MeHg bioaccumulation in marsh food webs, though this could increase MeHg transport to adjacent aquatic food webs.^61,80−82^ In addition, altering marsh hydrology could influence sediment biogeochemistry and reduce MeHg production by minimizing wetting and drying cycles to stabilize sediment redox conditions, by diluting concentrations of reactants such as sulfate and ferric iron that stimulate anaerobic microbial metabolism, or by enhancing MeHg dilution and removal processes.^82−84^ Such management approaches have been attempted but have achieved mixed results.^83,84^

Many physical, biogeochemical, and ecological processes influence the production, uptake, and biomagnification of MeHg in food webs. These processes can result in spatial variation in MeHg production and availability among marsh features, making it difficult to link MeHg production in sediments to bioaccumulation of MeHg in food webs. Our results indicated that sediment biogeochemistry differed among features with distinct hydrological and geomorphological characteristics, and these differences resulted in greater MeHg concentrations of sediments and surface water particulates from marsh interiors compared to other features. In contrast, model-predicted tissue MeHg concentrations of primary and secondary consumers differed among features, with greater concentrations in taxa from marsh plains (i.e., marsh edges and interiors) compared to channels. This spatial mismatch of MeHg bioaccumulation in consumers compared with MeHg concentrations in sediments and surface water particulates may have resulted from differences in behavior and physiology among consumer taxa that influenced the spatial scale over which MeHg was integrated into consumer tissues. These results suggested that remediation and restoration plans may reduce the potential for MeHg bioaccumulation in tidal marsh food webs by altering marsh hydrology, although additional studies are needed. Our results also highlight the importance of collecting samples across a suite of tidal marsh features and considering the behavioral and physiological traits of sentinel taxa for contaminant monitoring studies.

## Data Availability

Data from this research are archived at L.A.H., I.W., M.M.-D.P., Tsao, D. C., D.P.K., J.Y.T., and S.E.W.D.L.C. Sediment biogeochemistry and subsequent mercury biomagnification in wetland food webs of the San Francisco Bay, CA (ver. 2.0, November 2023); U.S. Geological Survey Data Release 2020. https://doi.org/10.5066/P9AMA3PL.

## Supporting Information Available

Materials and methods for sediment biogeochemical samples (Section 1); summary statistics of biogeochemical variables (Table S1); summary statistics of invertebrate and fish methylmercury concentrations (Table S2); study area map (Figure S1); tissue MeHg concentrations as a function of δ^15^N values (Figure S2); and tissue MeHg concentrations as a function of δ^13^C values (Figure S3) (PDF)
