# Supplementary material for: Linking Mesoscale Spatial Variation in Methylmercury Production to Bioaccumulation in Tidal Marsh Food Webs
Source: Environ Sci Technol. 2023 Nov 13;57(48):19263–73. doi: 10.1021/acs.est.3c04907 (PMC10702545; doi:10.1021/acs.est.3c04907)
Supplement: Supplementary file 1 — es3c04907_si_001.pdf [file es3c04907_si_001.pdf]

SUPPORTING INFORMATION

Linking meso-scale spatial variation in methylmercury production to bioaccumulation in tidal marsh food webs

Laurie A. Hall[1*], Isa Woo[1], Mark Marvin-DiPasquale[2], John Y. Takekawa[1,3], David P. Krabbenhoft[4], Donald Yee[5], Letitia Grenier[5], Susan E.W. De La Cruz[1]

[1]U.S. Geological Survey, Western Ecological Research Center, San Francisco Bay Estuary Field Station, NASA Research Park Bldg. 19, N. Akron Road, Moffett Field, CA 94035, USA

[2]U.S. Geological Survey, Water Mission Area, Earth System Processes Division, 345 Middlefield Road, Menlo Park, CA 94025, USA

[3]Current affiliation: Suisun Resource Conservation District, 2544 Grizzly Island Road, Suisun City, CA 94585, USA

[4]U.S. Geological Survey, Mercury Research Laboratory, 8505 Research Way, Middleton, WI 53562, USA

[5]San Francisco Estuary Institute, 4911 Central Avenue, Richmond, CA 94804, USA

*Corresponding author: Laurie Hall (lahall@usgs.gov)

Number of pages: 20

Number of tables: 2

Number of figures: 3

Supplemental Information Table of Contents

1. MATERIALS AND METHODS 3
1.1 Mercury Species in Sediment and Water 3
1.2 Microbial Rate Measurements in Sediment 4
1.3 Ancillary Bulk Sediment Assays 7
1.4 Ancillary Sediment Porewater Assays 9
2. TABLES 12
Table S1 12
Table S2 14
3. FIGURES 15
Figure S1 15
Figure S2 16
Figure S3 17
4. REFERENCES 18

1. MATERIALS AND METHODS

1.1 Mercury Species in Sediment and Water

Water samples were analyzed for methylmercury (MeHg) and sediment samples were analyzed for both MeHg and total mercury (THg) at the USGS Mercury Research Laboratory (Middleton, WI). Water samples were assayed for MeHg by distillation, aqueous ethylation, purge and trap, and cold vapor atomic fluorescence spectroscopy (CVAFS) as detailed in DeWild et al. (2002). Sediment THg samples were initially digested and oxidized with concentrated acid (aqua regia), followed by further oxidation with bromine monochloride at 50 °C (overnight), with the digestate quantified via $SnCl_2$ reduction, purge and trap, and CVAFS detection according to Olund et al. (2004). Sediment MeHg was initially extracted into methylene chloride and back extracted into reagent water, followed by aqueous ethylation, purge and trap, thermal desorption, GC separation, pyrolysis, and CVAFS detection, according to DeWild et al. (2004). Standard quality control samples were run with all THg and MeHg analytical batches. Digestion blanks using all reagents were employed through all the analytical steps and subtracted from the final result. At least 10% of all THg and MeHg analyses were run in replicate and agreed within ± 20% (acceptance criteria for the batches run). Standard reference material IAEA 405 (estuarine sediment, certified values: THg = 810 ng g^{-1}, MeHg = 5.49 ng g^{-1}) was used to ensure the accuracy of the analytical results, with data acceptance criteria within ±10% of the certified value.

Sediment reactive inorganic mercury (RHg) was assayed by the USGS Biogeochemistry Laboratory (Menlo Park, CA), and is an operationally defined proxy measure of the pool of inorganic Hg(II) available for Hg(II)-methylation that is based upon the readily tin-reducible fraction of THg in a bulk sediment sample (Marvin-DiPasquale and Cox, 2007; Marvin-

DiPasquale et al. 2009a, 2014). Previously sub-sampled and frozen sediment was thawed under anoxic conditions and slurried with anoxic 0.5 M HCl. The slurry was transferred to a gas purging bubbler and reacted with $SnCl_2$ for 15 minutes. The evolved elemental Hg^0 gas was captured on a gold trap, thermally desorbed, and measured via CVAFS (Marvin-DiPasquale et al. 2011). Bubbler blanks were run with all sample sets, with the blank values subtracted from each analytical result. All samples were run in duplicate. The relative percent deviation from the mean of analytical duplicate (n = 2) analyses was 12.3 ± 2.0% (mean ± standard error, n = 48 analytical pairs). There are no available certified reference materials for this assay.

1.2 Microbial Rate Measurements in Sediment

All microbial rate measurements were conducted by the USGS Biogeochemistry Laboratory (Menlo Park, CA). Sediment methylmercury production potential (MPP) rate constants (k_{meth}) were determined using laboratory bottle incubations. Composited sediment samples (surface 0-2 cm) collected in the field were stored chilled in mason jars with minimal to no headspace for approximately 24 hours before sub-sampling in an N_2 flushed anaerobic environment. Incubations were conducted within 48 hours of field collection. Sub-samples (3.0 g) of homogenized sediment from each site were incubated in duplicate for four hours after the addition of $^{203}HgCl_2$ (0.1 ml; specific activity adjusted to 1 μCi μg^{-1}; total Hg per sample = 500 ng g^{-1} wet sediment). Incubations were arrested by flash freezing samples on dry ice in ethanol. A single killed control (frozen at time = 0) was included with each site-specific specific set. Incubation times ranged from 5.0 to 5.9 hours and incubation temperature was set at 20 °C. The radiolabeled methylmercury ($Me^{203}Hg$) that was formed during the incubation was subsequently extracted with toluene and quantified via gamma radiation counting (Marvin-DiPasquale et al.

2003). Pseudo first-order values for k_{meth} were subsequently calculated as previously described (Marvin-DiPasquale and Agee 2003). The nominal detection limit for k_{meth} ranged from 0.00027 day^{-1} (April 2006) and 0.00072 day^{-1} (August 2006). The relative percent deviation from the mean of analytical duplicate (n = 2) kill corrected k_{meth} analyses was 38 ± 7% (mean ± standard error, n = 30 analytical pairs). MPP rates were calculated as the product of k_{meth} and the independently measured sediment RHg concentration (described above) as per Marvin-DiPasquale et al. (2008). This approach factors in both a measure of the activity of the native Hg(II)-methylating microbial community and a operationally defined measure of Hg(II) pool size that is available to that microbial community.

Microbial sulfate reduction (SR) rates in sediment were assayed via the $^{35}SO_4^{2-}$ radiotracer amendment technique (Jørgensen 1978). Sub-samples for SR consisted of 1.5 g of sediment per vial and were collected under anoxic conditions and incubated in parallel with those for k_{meth}. Replication consisted of duplicate live (incubated) and one killed control sample per site. Samples for SR were amended with approximately 1.0 µCi of carrier-free $^{35}SO_4^{2-}$ (0.05 ml of a 20 µCi ml^{-1} working stock of $Na_2{}^{35}SO_4$). Incubations were conducted at 20 °C for 5.0 to 5.9 hours and were arrested by the addition of 1 ml of 10% (w/v) zinc-acetate and subsequent freezing in an ethanol/dry ice bath. Upon thawing, total reduced sulfur (TRS) was extracted via distillation with an acidic chromium solution and measured for beta radioactivity (Fossing and Jørgensen 1989). Rate constants for SR were calculated as the fraction of ^{35}S-TRS produced, relative the amount of $^{35}SO_4^{2-}$ added, normalized by the incubation time. Nominal detection limits were 0.00047 day^{-1} (April 2006) and 0.00038 day^{-1} (August 2006). Rates of SR were then calculated from the site-specific rate constants and the *in situ* sediment porewater SO_4^{2-} concentration (Marvin-DiPasquale and Capone 1998). The relative percent deviation from the

mean of analytical duplicate (n = 2) kill corrected SR rates was 24 ± 3% (mean ± standard error, n = 42 analytical pairs).

Net rates of methane (CH_4) production (or loss) in sediment were measured from bottle incubations, which were conducted at 20 °C for 6 ± 0.1 days. Four sub-samples of homogenized bulk sediment (3.0 g, exact weight recorded) were collected for each sampling site under anoxic conditions (N_2 flushed glove bag), transferred into 13 ml serum bottles, stoppered and crimp sealed. Two of the four bottles per set were initially flash frozen in a dry ice and ethanol bath to arrest microbial activity (Day = 0 sample set). At the end of the six days, the second set of two bottles were similarly flash frozen (Day = 6 sample set). Subsequently, all of the incubation bottles were autoclaved to liberate the majority of the CH_4 from the bulk sediment into the bottle headspace (approx. 10 ml). Methane was measured via a gas chromatograph equipped with a flame ionization detector. Standards for calibration were prepared in a series of N_2 flushed serum bottles using serial dilutions of 99.99% pure CH_4 gas. Excess gas in the headspace of each bottle was measured using a wetted ground glass syringe. After accounting for any increased gas pressure in the bottle headspace and temperature, gaseous CH_4 concentration (in ppm) was converted to molar volume (nmol per liter) using the ideal gas law. The net daily change in CH_4 (production or loss) in the headspace was calculated by subtracting the mean (n = 2) of the Day = 0 pair from the mean (n = 2) of the Day = 6 pair and dividing by the total incubation time and by the original sediment dry mass (final units: nmol CH_4 per gram dry sediment per day).

Net daily rates of iron reduction (or oxidation; Fe(II) rate) in sediment were measured from the same set of bottle incubations that the CH_4 measurements were conducted in (described above). After measuring the CH_4 in the gas phase, 0.5 g of sediment from each incubation bottle was sub-sampled under anoxic conditions and assayed for acid-extractable ferrous iron (Fe(II),

see details below). The net daily change in Fe(II) was calculated by subtracting the mean (n = 2) of the Day = 0 pair from the mean (n = 2) of the Day = 6 pair and dividing by the total incubation time and by the sediment dry mass (final units: mg Fe(II) per gram dry sediment per day).

1.3 Ancillary Bulk Sediment Assays

All bulk sediment assays described below were conducted by the USGS Biogeochemistry Laboratory (Menlo Park, CA). Iron Speciation in bulk sediment samples followed the procedure and calculations detailed in Marvin-DiPasquale et al. (2008) and consisted of assaying three iron fractions, namely, acid extractable ferrous iron (Fe(II)), amorphous ferric iron ($Fe(III)_a$), and crystalline ferric iron ($Fe(III)_c$). The initial sub-sampling of sediment (1.00 ± 0.05 g per sample) was done under anoxic conditions in an N_2 flushed glove bag and samples were stored frozen until further processing using the method referenced above, with the Fe(II) and $Fe(III)_a$ fractions being run sequentially on the same sample and being assayed spectrophotometrically using Ferrozine (Lovley and Phillips, 1986). The $Fe(III)_c$ fraction was first extracted with dithionite-citrate before similarly being assayed spectrophotometrically using Ferrozine (Marvin-DiPasquale et al. 2008). Certified reference material for the various Fe-species is not commercially available. Calibration standards of $FeSO_4$ were prepared in a solution of 0.25 M hydroxylamine-HCl. The method detection limit is approximately 0.10 mg Fe g^{-1} dry sediment, as processed. Commercially Fe_2O_3 (hematite) was used as matrix spike material for the $Fe(III)_c$ fraction, which had a mean percent recovery of 90 ± 15% (n = 5). No commercially available Fe(III) material was found to be appropriate for use as an 'amorphous ferric iron' (that is, $Fe(III)_a$) matrix spike. For the current study, the relative percent deviation from the mean of analytical duplicate (n = 2) samples for each of the three iron fractions (mean ± standard error,

[*n*] analytical pairs) was: Fe(II), 8.5 ± 1.2% [48]); Fe(III)$_a$, 17 ± 2% [48]; and Fe(III)$_c$, 36 ± 14% [40].

Bulk sediment acid volatile sulfur (AVS) was quantified using a modified acid distillation approach (Zhabina and Volkov 1978) as described in greater detail in Marvin-DiPasquale et al. (2008). Upon sub-sampling, 1.0-1.5 g of homogenized whole sediment was accurately weighed (± 0.01 g) and transferred into a 10 ml serum vial, under anoxic conditions. Sub-samples were preserved with the addition of 5.0 ml of anoxic 10% (w/v) zinc-acetate solution and stored frozen (-20 °C) until further analysis. Upon partial thawing, the sample was distilled under anoxic conditions in an acidic solution of titanium chloride. The liberated H_2S gas was trapped as ZnS precipitate in a 10 ml solution of 10% (w/v) zinc acetate. The ZnS precipitate solution was subsequently subsampled in duplicate and quantified by colorimetric analysis (Cline 1969). A ZnS primary stock solution was used to prepare both a calibration curve (via serial dilution) for the S^{2-} colorimetric assay and for distillation efficiency tests (ZnS spike added to distillation rig without sediment). The mean distillation recovery (mean ± standard error) was 95 ± 6% (n = 15). The relative percent deviation from the mean of analytical duplicate (n = 2) AVS samples was 19 ± 2% (mean ± standard error, n = 48 analytical pairs).

Bulk sediment total reduced sulfur (TRS) was quantified on the same set of samples that the microbial SR measurements (described above) were made, and thus included results from the two live and one killed control sample (per set). TRS was extracted via distillation with a heated acidic chromium solution (Fossing and Jørgensen 1989), as detailed in Marvin-DiPasquale et al. (2008). Like the AVS assay, a ZnS primary stock solution was used to prepare both a calibration curve (via serial dilution) for the S^{2-} colorimetric assay and for distillation efficiency tests (ZnS spike added to distillation rig without sediment). The mean distillation recovery (mean ±

standard error) was 72 ± 7% (n = 8). The relative percent deviation from the mean of analytical duplicate (n = 2) TRS samples was 20 ± 2% (mean ± standard error, n = 46 analytical pairs).

Bulk sediment measurements of weight loss on ignition (LOI), percent of wet weight (DW), bulk density (BD), and porosity (POR) were all made on the same sample, as detailed in Marvin-DiPasquale et al. (2008). For the current study, the relative percent deviation from the mean of analytical duplicate (n = 2) samples for each of these sediment assays was (mean ± standard error, [n] analytical pairs): LOI, 4.5 ± 0.6% [46]); percent of wet weight, 1.5 ± 0.3% [46]; bulk density, 2.0 ± 0.3% [46]; and porosity, 1.7 ± 0.3% [46].

Bulk sediment pH and redox (oxidation-reduction potential; Eh) were measured by electrochemical probe directly inserted into homogenized sediment, as detailed in Marvin-DiPasquale et al. (2008; see subsection entitled: *Ancillary Sediment Measures Associated with Composite Samples Collected for Microbial Rate Assays*). Only a single measurement for each analyte was made per sample set.

Bulk sediment grain size (GS; greater or less than 63 microns, the sand/silt split) was assayed using a standard wet sieve method (Matthes et al. 1992). The relative percent deviation from the mean of analytical duplicate (n = 2) grain size samples was 3.3 ± 0.4% (mean ± standard error, n = 48 analytical pairs).

1.4 Ancillary Sediment Porewater Assays

All sediment porewater assays described below were conducted by the USGS Biogeochemistry Laboratory (Menlo Park, CA). Porewater was collected from each composite sediment sample under oxygen-free conditions by centrifugation followed by filtration filtered through a 0.45 µm syringe filter. Preservation for each porewater fraction are identified below.

Porewater dissolved organic carbon (pwDOC) samples were initially preserved by freezing and subsequently assayed using high temperature combustion and IR (infrared) detection on a Total Organic Carbon Analyzer (Model TOC-VCPH, Shimadzu Scientific Instruments, Columbia, Md.). Quality assurance measures include calibration standards, laboratory reagent blank, and filter blanks. The method detection limit was approximately 0.2 mg C L^{-1}. The relative percent deviation from the mean of analytical duplicate (n = 2) pwDOC samples was 5.8 ± 1.0% (mean ± standard error, n = 40 analytical pairs).

Porewater acetate (pwAcetate) samples were initially preserved by freezing and subsequently assayed on a HPLC (Shimadzu, Model 10A, VP series) equipped with a BioRad Aminex HPX-87H analytical column and a UV-visual detector (SPD-10AVP) set at 210 nm. The mobile phase (2 ml/minute) consisting of 0.016 N H_2SO_4. Quality assurance included calibration standards prepared from concentrated commercial stock solutions, laboratory reagent blanks, filter blanks, analytical duplicates and matrix spikes. The reporting limit and method detection limit was approximately 24 µmol L^{-1} and 5 µmol L^{-1}, respectively. The matrix spike recovery was 111 ± 9% (n = 2). The relative percent deviation from the mean of analytical duplicate (n = 2) porewater acetate samples was 24% (n = 1 analytical pair).

Porewater ferrous iron (pwFe(II)) samples were initially preserved with HNO_3 (pH < 2) and stored refrigerated prior to being assayed via colorimetric analysis (Marvin-DiPasquale et al., 2008). Quality assurance measures include calibration standards, laboratory reagent blank, and duplicate analyses. The detection limit for this assay was approximately 0.02 µg mL^{-1} at the level of the spectrophotometric analysis. The relative percent deviation from the mean of analytical duplicate (n = 2) pwFe(II) samples was 17 ± 3% (n = 38 analytical pairs).

Porewater sulfide (pwH_2S) samples were initially preserved 1:1 with anoxic sulfur antioxidant buffer (SAOB) and stored refrigerated prior to being assayed via ion selective electrode potentiometric quantification (Marvin-DiPasquale et al., 2009b). Quality assurance included calibration standards, reagent blanks, and analytical duplicates. The detection limit for this assay was approximately 0.3 μmol L^{-1}. The relative percent deviation from the mean of analytical duplicate (n = 2) porewater sulfide samples was 14 ± 2% (n = 41 analytical pairs).

Porewater sulfate ($pwSO_4^{2-}$) and chloride ($pwCl^-$) were measured on the same sub-sample via ion chromatography, with samples being initially preserved frozen prior to being assayed (Marvin-DiPasquale et al., 2009b). Quality assurance included calibration standards, laboratory reagent blanks, filter blanks, and analytical duplicates. The reporting limits for sulfate and chloride were approximately 5 μmol L^{-1} and 14 μmol L^{-1}, respectively, at the level of the detector. The relative percent deviation from the mean of analytical duplicate (n = 2) porewater sulfate and chloride samples was 5.3 ± 0.9% (n = 41 analytical pairs) and 4.7 ± 1.1% (n = 39 analytical pairs), respectively.

2. TABLES

Table S1. Means (and ranges) of 26 biogeochemical variables measured in sediments and porewater of four marsh features: marsh edge, marsh interior, first order channels, and third order channels collected at tidal marshes along the Petaluma River in California, USA. Variables included: total mercury (THg), inorganic reactive mercury (RHg), methylmercury (MeHg), MeHg production rate constant (K_{meth}), MeHg production potential (MPP), percent organic matter (LOI), porewater dissolved organic carbon (pwDOC), porewater acetate (pwAcetate), sediment redox (Eh), pH, grain size (GS), percent of wet weight (DW), bulk density (BD), porosity (POR), microbial sulfate reduction rate (SR), acid volatile sulfur (AVS), total reduced sulfur (TRS), porewater sulfide (pwH_2S), porewater sulfate ($pwSO_4^{2-}$), porewater chloride ($pwCl^-$), microbial iron reduction rate (Fe(II) rate), acid extractable ferrous iron (Fe(II)), amorphous ferric iron ($Fe(III)_a$), crystalline ferric iron ($Fe(III)_c$), porewater ferrous iron (pwFe(II)), and methane production (CH_4 prod.).

Variable (units)	Marsh edge	Marsh interior	1st order channels	3rd order channels
Mercury content				
THg (ng g^{-1} dry wt.)	333.3 (311.1-356.1)	254.0 (171.6-296.7)	280.0 (236.5-311.9)	375.8 (323.2-430.6)
RHg (ng g^{-1} dry wt.)	6.3 (2.1-11.7)	0.8 (0.1-2.2)	1.0 (0.1-6.0)	1.0 (0.1-5.2)
MeHg (ng g^{-1} dry wt.)	2.2 (0.4-5.6)	5.1 (0.4-14.5)	1.2 (0.3-3.6)	1.7 (0.6-4.1)
k_{meth} (1 d^{-1})	0.002 (0.0003-0.009)	0.1 (0.002-0.3)	0.02 (0.0003-0.08)	0.005 (0.0003-0.01)
MPP (pg g^{-1} d^{-1} dry wt.)	13.2 (1.5-33.8)	43.8 (4.9-147.3)	5.9 (0.08-26.7)	2.9 (0.09-9.8)
Organic matter				
LOI (% of dry wt.)	11.4 (9.8-13.5)	20.3 (11.6-31.7)	11.2 (8.7-14.1)	8.0 (6.4-10.0)
pwDOC (mg L^{-1})	98.0 (38.0-181.4)	95.4 (45.0-174.8)	26.8 (15.0-62.0)	24.9 (15.3-59.0)
pwAcetate (µmol L^{-1})	250 (45-483)	210 (0-525)	21 (0-78)	41 (0-237)
Sediment characteristics				
E_h (Mv)	216 (154-322)	70 (-67-213)	50 (-45-192)	68 (-31-198)
pH (standard units)	6.5 (5.8-6.9)	6.9 (6.5-7.4)	7.1 (6.8-7.3)	7.3 (7.0-7.6)
GS (% < 64 µm)	79.0 (57.4-89.4)	75.9 (63.8-89.8)	66.8 (57.6-75.2)	72.2 (58.5-80.5)
DW (% of wet wt.)	47.7 (41.5-54.6)	28.2 (19.4-35.3)	33.2 (28.9-39.0)	39.1 (35.0-43.2)
BD (g cm^{-3} wet sed.)	1.3 (1.1-1.5)	1.1 (1.1-1.2)	1.2 (1.1-1.3)	1.3 (1.1-1.5)
POR (ml PW cm^{-3} wet sed.)	0.7 (0.6-0.7)	0.8 (0.7-0.9)	0.8 (0.7-0.8)	0.8 (0.7-1.0)

Table S1 cont.

Sulfur speciation and microbial sulfate reduction				
SR (μmol g^{-1} dry sed. D^{-1})	0.1 (0.002-0.4)	2.6 (0-13.5)	0.4 (0.05-1.8)	0.2 (0.004-1.1)
AVS (μmol g^{-1} dry wt.)	1.2 (0.1-3.2)	37.6 (3.1-82.6)	77.5 (1.5-184.4)	29.0 (1.7-94.2)
TRS (μmol g^{-1} dry wt.)	27 (12-54)	84 (12-213)	203 (23-483)	174 (18-568)
pwH_2S (μmol L^{-1})	3.9 (1.1-8.7)	8.2 (3.0-24.2)	3.4 (1.1-9.9)	3.9 (1.1-11.1)
pwSO_4^{2-} (mmol L^{-1})	7.5 (0-22.1)	10.2 (1-21.4)	8.4 (0-16.4)	8.2 (1-19.4)
pwCl^- (mmol L^{-1})	147 (4-338)	240 (39-421)	191 (37-364)	169 (19-392)
Iron speciation and microbial iron reduction				
Fe(II) rate (mg g^{-1} dry sed. D^{-1})	0.2 (-0.01-0.6)	0.08 (-0.9-0.9)	0.4 (-0.3-0.9)	0.1 (-0.1-0.5)
Fe(II) (mg g^{-1} dry wt.)	1.3 (0.1-3.3)	6.2 (2.3-10.3)	12.2 (1.0-21.7)	8.6 (1.2-19.1)
Fe(IIIa) (mg g^{-1} dry wt.)	3.0 (2.3-3.9)	2.2 (0.2-6.2)	10.1 (0.8-25.8)	3.5 (0.4-9.4)
Fe(IIIc) (mg g^{-1} dry wt.)	10.9 (8.1-12.9)	4.6 (0.1-12.2)	6.9 (0.1-21.2)	8.8 (0.8-20.5)
pwFe(II) (mg L^{-1})	0.7 (0.6-2.6)	5.1 (0.2-27.5)	5.0 (0.1-11.2)	1.8 (0.04-7.1)
Methanogenesis				
CH_4 Prod. (nmol g^{-1} dry sed. D^{-1})	10.9 (-0.4-59.8)	2.3 (-4.9-12.3)	6.9 (-3.0-58.5)	0.4 (-0.4-2.3)

Table S2. Means, standard errors, minimums, maximums, and sample sizes (*N*) for methylmercury concentrations (µg g^{-1}) of invertebrate and fish consumers in tidal marsh food webs collected at three marshes: Black John Slough, Mid-Petaluma Marsh, and Gambinini Marsh, along the Petaluma River in California, USA. Standard errors were not estimated for taxa with less than three samples.

Taxon	Sample type	*N*	Mean	St. error	Minimum	Maximum
Assiminea sp.	Invertebrate	6	0.10	0.01	0.07	0.14
Myosotella myosotis	Invertebrate	6	0.15	0.02	0.10	0.23
Traskorchestia sp.	Invertebrate	12	0.13	0.01	0.09	0.23
Bembidion sp.	Invertebrate	2	0.46	NA	0.32	0.60
Pardosa sp.	Invertebrate	11	0.50	0.04	0.30	0.74
Geukensia demissa	Invertebrate	19	0.24	0.02	0.13	0.53
Macoma petalum	Invertebrate	11	0.14	0.02	0.06	0.30
Palaemon sp.	Invertebrate	3	0.04	0.02	0.01	0.07
Hemigrapsus sp.	Invertebrate	6	0.56	0.28	0.08	1.89
Tridentiger bifasciatus	Fish	12	0.33	0.03	0.19	0.44
Acanthogobius flavimanus	Fish	5	0.24	0.05	0.15	0.42
Gillichthys mirabilis	Fish	1	0.44	NA	0.44	0.44
Gambusia affinis	Fish	5	0.58	0.18	0.23	1.25
Gasterosteus aculeatus	Fish	16	0.58	0.07	0.32	1.43
Cottus gulosus	Fish	1	0.28	NA	0.28	0.28

3. FIGURES

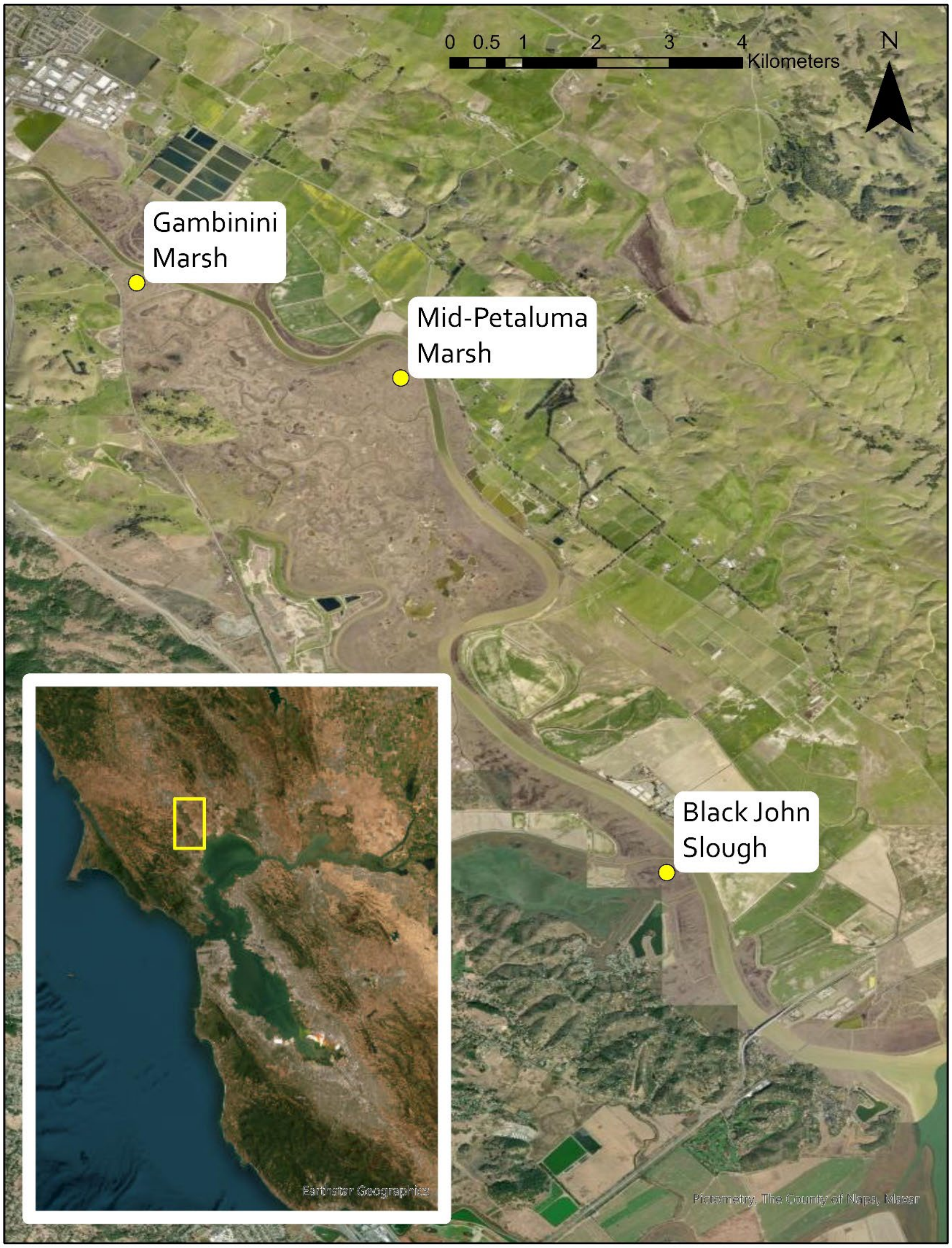

Figure S1. Map of three wetland sites: Black John Slough, Mid-Petaluma Marsh, and Gambinini Marsh, along the Petaluma River in northern San Francisco Bay, CA, USA.

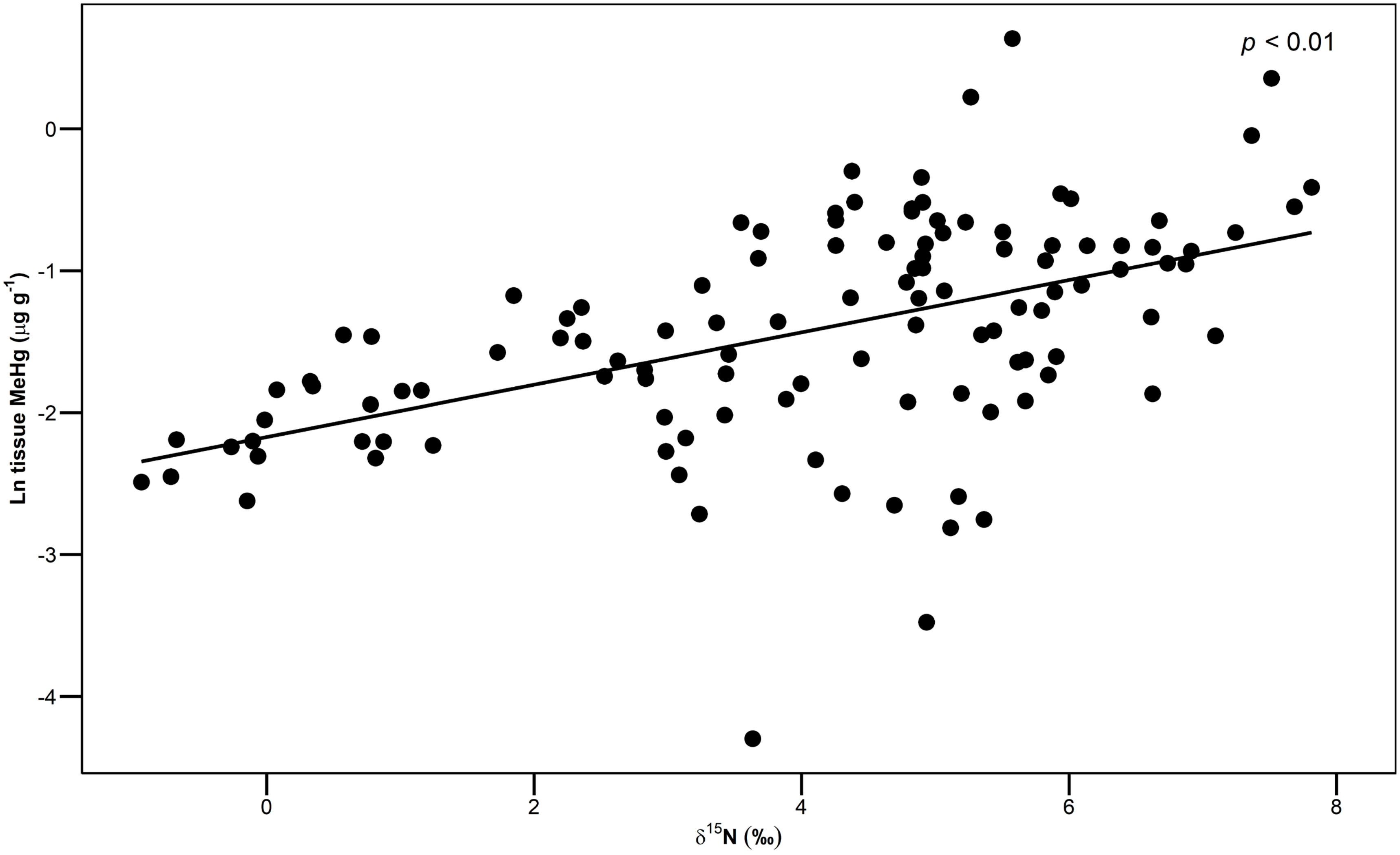

Figure S2. Natural-log-transformed tissue methylmercury (MeHg) concentrations of primary and secondary consumers as a function of stable δ^{15}N (baseline corrected) isotope values from tidal marsh food webs collected at three marshes: Black John Slough, Mid-Petaluma Marsh, and Gambinini Marsh, along the Petaluma River in California, USA. Statistical significance was assessed with a general linear model that included δ^{15}N, δ^{13}C, site, and marsh feature as predictor variables.

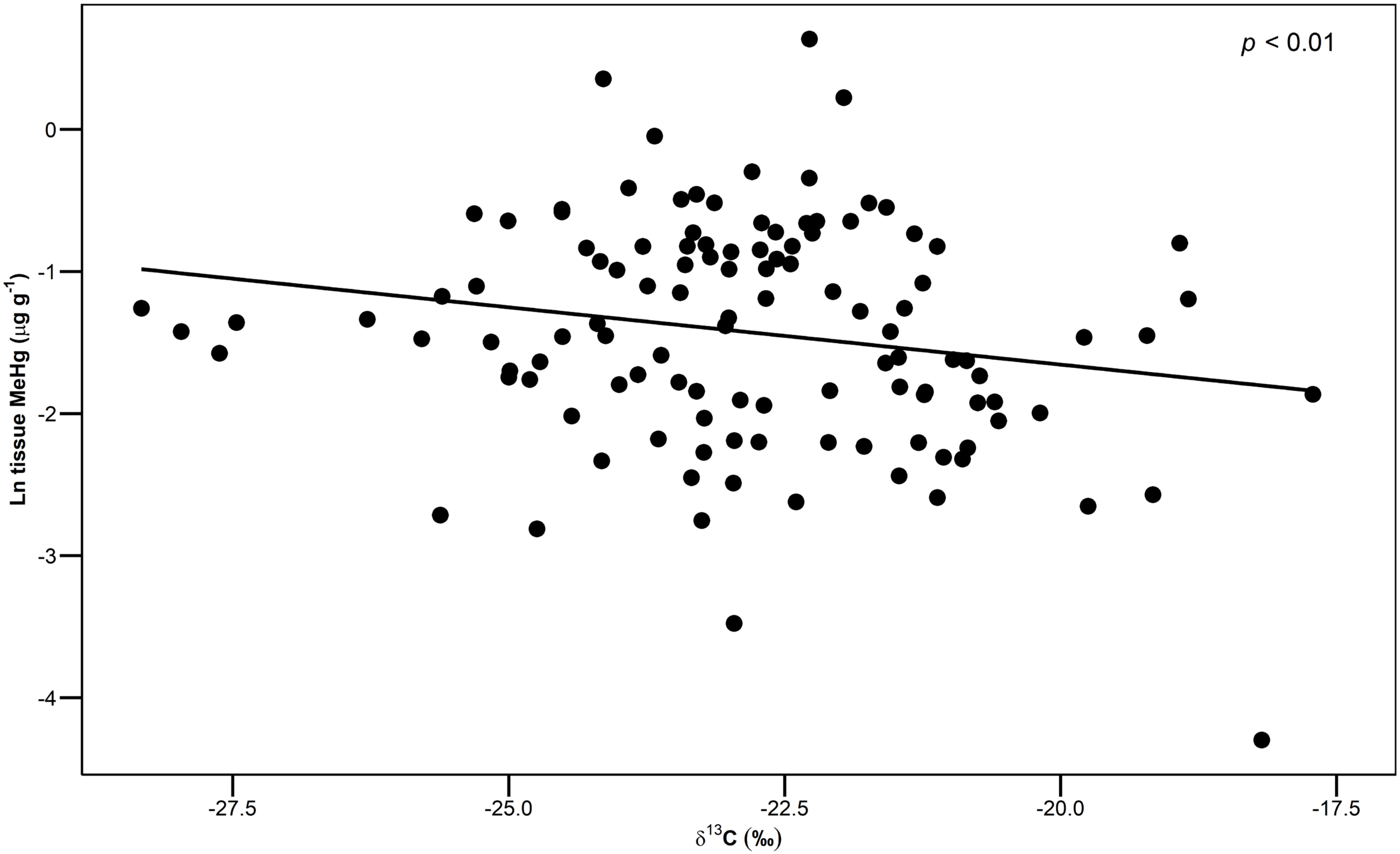

Figure S3. Natural-log-transformed tissue methylmercury (MeHg) concentrations of primary and secondary consumers as a function of stable $\delta^{13}C$ (normalized for variable lipid content) isotope values from tidal marsh food webs collected at three marshes: Black John Slough, Mid-Petaluma Marsh, and Gambinini Marsh, along the Petaluma River in California, USA. Statistical significance was assessed with a general linear model that included $\delta^{15}N$, $\delta^{13}C$, site, and marsh feature as predictor variables.

4. REFERENCES

Cline, J. D. (1969). Spectrophotometric determination of hydrogen sulfide in natural waters: *Limnology and Oceanography*, 14: 454-458.

DeWild, J. F., M. L. Olson, and S. D. Olund (2002). Determination of Methyl Mercury by Aqueous Phase Ethylation, Followed by Gas Chromatographic Separation with Cold Vapor Atomic Fluorescence Detection. U. S. Geological Survey Open File Report 01-445, 19 p., https://doi.org/10.3133/ofr2001445

DeWild, J. F., S. D Olund, M. L. Olson, and M. T. Tate (2004). Methods for the Preparation and Analysis of Solids and Suspended Solids for Methylmercury. U. S. Geological Survey Techniques and Methods 5 A–7, http://pubs.usgs.gov/tm/2005/tm5A8/

Fossing, H., and B. Jørgensen (1989). Measurement of bacterial sulfate reduction in sediments: Evaluation of a single step chromium reduction method. *Biogeochemistry*, 8: 205-222.

Jørgensen, B. B. (1978). A comparison of methods for the quantification of bacterial sulfate reduction in coastal marine sediments. *Geomicrobiology Journal*, 1(1): 11-27.

Lovley, D. R., and E. J. P. Phillips (1986). Organic matter mineralization with reduction of ferric iron in anaerobic sediments. *Applied and Environmental Microbiology*, 51(4): 683-689.

Marvin-DiPasquale, M. C., and D. G. Capone (1998). Benthic sulfate reduction along the Chesapeake Bay central channel. I. Spatial trends and controls. *Marine Ecology Progress Series*, 168: 213-228.

Marvin-DiPasquale, M., and J. L. Agee (2003). Microbial mercury cycling in sediments of the San Francisco Bay-Delta. *Estuaries*, 26(6): 1517-1528.

Marvin-DiPasquale, M., J. Agee, R. Bouse, and B. Jaffe (2003). Microbial cycling of mercury in contaminated pelagic and wetland sediments of San Pablo Bay, California. *Environmental Geology*, 43(3): 260-267.

Marvin-DiPasquale, M., and M. H. Cox (2007). Legacy mercury in Alviso Slough, South San Francisco Bay, California—Concentration, speciation and mobility. U. S. Geological Survey Open-File Report number 2007-1240, 98 p., http://pubs.usgs.gov/of/2007/1240/.

Marvin-DiPasquale, M. C., M. A. Lutz, D. P. Krabbenhoft, G. R. Aiken, W. H. Orem, B. D. Hall, J. F. DeWild, and M. E. Brigham (2008). Total Mercury, Methylmercury, Methylmercury Production Potential, and Ancillary Streambed-Sediment and Pore-Water Data for Selected Streams in Oregon, Wisconsin, and Florida, 2003-04: U. S. Geological Survey Data Series 375, 25 p., http://pubs.er.usgs.gov/usgspubs/ds/ds375

Marvin-DiPasquale, M., M. A. Lutz, M. E. Brigham, D. P. Krabbenhoft,G. R. Aiken, W. H. Orem, and B. D. Hall (2009a). Mercury Cycling in Stream Ecosystems. 2. Benthic Methylmercury Production and Bed Sediment−Pore Water Partitioning. *Environmental Science & Technology*, 43(8): 2726-2732.

Marvin-DiPasquale, M., C. N. Alpers, and J. A. Fleck (2009b). Mercury, methylmercury, and other constituents in sediment and water from seasonal and permanent wetlands in the Cache Creek Settling Basin and Yolo Bypass, Yolo County, California, 2005−06: U. S. Geological Survey, Open File Report 2009-1182, 69 p., http://pubs.usgs.gov/of/2009/1182/

Marvin-DiPasquale, M., J. L. Agee, E. Kakouros, L. H. Kieu, J. A. Fleck, and C. N. Alpers (2011). The Effects of Sediment and Mercury Mobilization in the South Yuba River and Humbug Creek Confluence Area, Nevada County, California: Concentrations, Speciation and Environmental Fate — Part 2, Laboratory Experiments: U. S. Geological Survey Open-File Report 2010-1325-B, 53 p., http://pubs.usgs.gov/of/2010/1325B

Marvin-DiPasquale, M., L. Windham-Myers, J. L. Agee, E. Kakouros, L. H. Kieu, J. Fleck, C. N. Alpers, and C. Stricker (2014) Methylmercury production in sediment from agricultural

and non-agricultural wetlands in the Yolo Bypass, California. *Science of the Total Environment*, (484): 288-299.

Matthes, W. J. J., C. J. Sholar, and J. R. George (1992) Quality-Assurance Plan for the Analysis of Fluvial Sediment by Laboratories of the U.S. Geological Survey: U. S. Geological Survey, Open-File Report 91-467, 37 p., https://doi.org/10.3133/ofr91467

Olund, S. D., J. F. DeWild, M. L. Olson, and M. T. Tate (2004) Methods for the preparation and analysis of solids and suspended solids for total mercury. Chapter 8 of Book 5, Laboratory Analysis; Section A, Water Analysis: U. S. Geological Survey, Reston USGS Techniques and Methods Report 5 A 8, 23 p., http://pubs.er.usgs.gov/usgspubs/tm/tm5A8

U.S. Environmental Protection Agency (2002). Method 1631, Revision E: Mercury in water by oxidation, purge and trap, and cold vapor atomic fluorescence spectrometry. U. S. Environmental Protection Agency, Office of Water, Washington D. C.: EPA-821-R-02-019, 36 pp.

Zhabina, N., and Volkov, I. (1978). A method of determination of various sulfur compounds in sea sediment and rocks, *In:* Krumbein, W.E., ed., Environmental Biogeochemistry and Geomicrobiology, Vol. 3: Methods, Metals, and Assessment: Ann Arbor, Ann Arbor Science Publishers, p. 735-746.
